# Improving the outcomes of foot and ankle surgery. Professional impact of the Australasian College of Podiatric Surgeons' audit tool

**DOI:** 10.1186/1757-1146-8-S2-O18

**Published:** 2015-09-22

**Authors:** Rob Hermann, Renata Meuter, Paul Bennett

**Affiliations:** 1 School of Psychology and Counselling, Queensland University of Technology (QUT), Kelvin Grove, 4059, Australia; 2 School of Clinical Science, QUT, Kelvin Grove, 4059, Australia

**Keywords:** Audit, surgery, consensus

## Background

Surgery is an example of expanded practice scope that enhances podiatry and incorporates inter-professional collaboration. By 2050 demand for foot and ankle procedures is predicted to rise nationally by 61.9%. Performance management of this increase motivated the development of an online audit tool. Developed in collaboration with the Australasian College of Podiatric Surgeons (ACPS), the ACPS audit tool provides real-time data capture and reporting. It is the first audit tool designed in Australia to support and improve the outcomes of foot and ankle surgery.

## Methods

Audit activity in general, orthopaedic, plastic and podiatric surgery was examined using a case study design. Audit participation enablers and barriers were explored. Case study results guided a Delphi survey of international experts experienced or associated with foot and ankle surgery. Delphi survey-derived consensus informed modification of a generic data set from the Royal Australasian College of Surgeons (RACS). Based on the Delphi survey findings the ACPS online audit tool was developed and piloted. Reliability and validity of data entry and usability of this new tool was then assessed with an online survey.

## Results

The case study found surgeon attitudes and behaviours positively impacted audit participation, and also indicated that audit data should be (1) available in real time, (2) identify practice change, (3) applicable for safety and quality management, and (4) useful for peer review discussion. The Delphi process established consensus on audit variables to be captured, including the modified RACS generic data set. 382 cases of foot and ankle surgery were captured across 3 months using the new tool. Data entry was found to be valid and reliable. Real-time outcome reporting and practice change identification impacted positively on safety and quality management and assisted peer review discussion. An online survey showed high levels of usability.

## Conclusions

Surgeon contribution to audit tool development resulted in 100% audit participation. The data from the ACPS audit tool supported the ACPS submission to the Medical Services Advisory Committee to list podiatric surgery under Medicare, an outcome noted by the Federal Minister of Health.

